# Promoter hypermethylation of *SHOX2* and *SEPT9* is a potential biomarker for minimally invasive diagnosis in adenocarcinomas of the biliary tract

**DOI:** 10.1186/s13148-016-0299-x

**Published:** 2016-12-12

**Authors:** V. Branchi, P. Schaefer, A. Semaan, A. Kania, P. Lingohr, J. C. Kalff, N. Schäfer, G. Kristiansen, D. Dietrich, H. Matthaei

**Affiliations:** 1 Department of General, Visceral, Thoracic and Vascular Surgery University Hospital Bonn, University of Bonn, Sigmund-Freud-Strasse 25, 53127 Bonn, Germany; 2 Institute of Pathology, University Hospital Bonn, Bonn, Germany; 3 Department of Otolaryngology, Head and Neck Surgery, University Hospital Bonn, Bonn, Germany

## Abstract

**Background:**

Biliary tract carcinoma (BTC) is a fatal malignancy which aggressiveness contrasts sharply with its relatively mild and late clinical presentation. Novel molecular markers for early diagnosis and precise treatment are urgently needed. The purpose of this study was to evaluate the diagnostic and prognostic value of promoter hypermethylation of the *SHOX2* and *SEPT9* gene loci in BTC.

**Methods:**

Relative DNA methylation of *SHOX2* and *SEPT9* was quantified in tumor specimens and matched normal adjacent tissue (NAT) from 71 BTC patients, as well as in plasma samples from an independent prospective cohort of 20 cholangiocarcinoma patients and 100 control patients. Receiver operating characteristic (ROC) curve analyses were performed to probe the diagnostic ability of both methylation markers. DNA methylation was correlated to clinicopathological data and to overall survival.

**Results:**

*SHOX2* methylation was significantly higher in tumor tissue than in NAT irrespective of tumor localization (*p* < 0.001) and correctly identified 71% of BTC specimens with 100% specificity (AUC = 0.918; 95% CI 0.865–0.971). *SEPT9* hypermethylation was significantly more frequent in gallbladder carcinomas compared to cholangiocarcinomas (*p* = 0.01) and was associated with large primary tumors (*p* = 0.01) as well as age (*p* = 0.03). Cox proportional hazard analysis confirmed microscopic residual tumor at the surgical margin (R1-resection) as an independent prognostic factor, while *SHOX2* and *SEPT9* methylation showed no correlation with overall survival. Elevated DNA methylation levels were also found in plasma derived from cholangiocarcinoma patients. *SHOX2* and *SEPT9* methylation as a marker panel achieved a sensitivity of 45% and a specificity of 99% in differentiating between samples from patients with and without cholangiocarcinoma (AUC = 0.752; 95% CI 0.631–0.873).

**Conclusions:**

*SHOX2* and *SEPT9* are frequently methylated in biliary tract cancers. Promoter hypermethylation of *SHOX2* and *SEPT9* may therefore serve as a minimally invasive biomarker supporting diagnosis finding and therapy monitoring in clinical specimens.

**Electronic supplementary material:**

The online version of this article (doi:10.1186/s13148-016-0299-x) contains supplementary material, which is available to authorized users.

## Background

Biliary tract cancers (BTC) comprise aggressive neoplasms arising from the epithelial lining of the intra- and extrahepatic bile ducts as well as the gallbladder. Cholangiocarcinoma (CC) and gallbladder carcinoma (GBC) account for only about 3% of all gastrointestinal malignancies [1]. Nonetheless, during the last decades, a steady increase in incidence and mortality rates has been reported for BTC in Europe as well as in the USA [2, 3]. This relatively uncommon malignancy is associated with an overall poor prognosis. In contrast to other cancer entities (e.g., breast, prostate, lymphoma), the 5-year survival rate of BTC has hardly changed during the last years and is not exceeding the 20% threshold [4–6]. Complete surgical resection with negative histological margins is the only potentially curative therapy to date though most patients rapidly develop recurrence even after R0 resection. Clinical symptoms normally appear late and most patients are therefore diagnosed in an advanced stage when a radical surgical resection is no longer possible [7]. Merely palliative treatment can be offered to patients with irresectable BTC [7, 8]. Even high resolution imaging such as computed tomography (CT) and magnetic resonance imaging (MRI) are sometimes inaccurate. Furthermore, advanced endoscopic techniques including endoscopic ultrasonography fine needle aspiration (EUS-FNA) biopsy or brush cytology harvested within endoscopic retrograde cholangiography (ERC) not always lead to conclusive diagnoses [9, 10]. Moreover, established serum biomarkers for BTC like CA 19-9 and CEA have limited sensitivity and specificity in disease detection and response prediction [9, 11].

There is emerging evidence that genetic and epigenetic alterations play a pivotal role during human carcinogenesis [12, 13]. Specific DNA methylation patterns ensure proper gene expression, thereby regulating various fundamental organic processes, such as cellular differentiation and development [14]. Accordingly, aberrant methylation of CpG dinucleotides has been evidenced as a hallmark epigenetic alteration in numerous malignant diseases [14, 15]. Promoter hypermethylation, in particular, has been linked to transcriptional silencing of tumor suppressor genes, thus enabling neoplastic cells to proliferate without restriction [15]. In BTC and its precursor lesions, these epigenetic alterations can be detected with possible valuable clinical implications [16–29].

The short stature homeobox 2 gene (*SHOX2*) is located on chromosome 3q25 → 3q26.1 [30]. As a transcriptional factor, *SHOX2* is known to be involved in various embryonic developmental processes including limb formation and cardiac development [31–33]. Aberrant DNA methylation of *SHOX2* has been extensively characterized as a biomarker for the diagnosis of lung cancer. DNA from lung cancer specimens appeared to be significantly higher methylated at the *SHOX2* gene locus as compared to morphologically normal adjacent tissue, and a correlation of hypermethylation and gene amplification has been described [34]. Thus, *SHOX2* methylation has been suggested as a biomarker in body fluids, e.g., blood plasma, bronchial aspirates, ascites, and pleural effusions [35–41]. Accordingly, a commercially available in vitro diagnostic test kit, the Epi proLung® BL Reflex Assay (Epigenomics AG, Berlin, Germany) has been developed for sensitive assessment of *SHOX2* methylation in bronchial aspirates [38]. Recently, increased *SHOX2* methylation levels were also detected in metastatic lymph node tissue obtained by endobronchial ultrasound with transbronchial needle aspiration (EBUS-TBNA) performed in patients with lung cancer, further improving the assessment of nodal status and staging in this entity [42].

Septins are a family of GTP-ase proteins which play a pivotal role in the cell cycle and cytokinesis [43, 44]. *SEPT9*, a member of the Septin gene family, has been reported to exhibit both oncogenic and tumor suppressive properties in tumorigenesis of solid and hematological malignancies [45]. DNA methylation of *SEPT9* has been widely studied in colorectal cancer (CRC). Tumor DNA exhibited significantly higher *SEPT9* methylation levels in comparison with matched normal colon epithelium [46, 47]. Hypermethylation of *SEPT9* was also present in colon adenomas, thus indicating that this epigenetic alteration is an early event in the adenoma-carcinoma sequence [47]. Methylated *SEPT9* has also been detected in plasma samples from colorectal cancer patients, and several studies have proposed its biomarker application for early diagnosis of CRC [46, 48–50]. Furthermore, *SEPT9* was found to be frequently methylated in the head and neck and esophageal squamous cell carcinoma, as well as prostate cancer [51–53]. Recently, a panel of four DNA methylation markers including *SEPT9* has been reported to aid in the diagnosis of cholangiocarcinoma in tissue as well as biliary brush samples [16].

Methylation status of *SHOX2* and *SEPT9* and their possible clinical implication has hardly been investigated in BTC. Due to the encouraging results found in other cancer entities, we hypothesize that these epigenetic alterations can serve as biomarkers in cancers arising from the biliary tract. Our long-term goal is to identify novel biomarkers for diagnostic and prognostic purposes to support and individualize treatment of this fatal disease.

## Methods

This study was conducted with approval of the Institutional Review Board (IRB) at the University of Bonn, Germany.

### Tissue samples

A total of 71 patients who underwent surgical resection for intrahepatic (IHC), perihilar (PHC), or distal cholangiocarcinoma (DC) as well as gallbladder carcinoma (GBC) at the Department of Surgery, University of Bonn, between January 1st, 1990, and December 31st, 2012, were enrolled. Inclusion criteria were (a) histological diagnosis of adenocarcinoma of the biliary tract (BTC); (b) primary resection of the tumor; (c) R0- or R1-resection; and (d) availability of archival formalin-fixed paraffin-embedded tissue blocks (Institute of Pathology, University of Bonn). All BTCs were staged according to the current edition of the American Joint Committee on Cancer (AJCC) cancer staging manual [54]. Relevant demographic and clinicopathological data were catalogued. Information on overall survival was obtained during follow-up visits in the outpatient clinic of our department, or by contacting patients or the general practitioners.

Hematoxylin and eosin slides were routinely prepared from FFPE tissue sections and reviewed under a light microscope. For each patient, an area with the highest tumor cellularity was selected, and a punch biopsy of 1-mm diameter was taken from the respective FFPE block. A second punch biopsy representing morphologically normal adjacent tissue (NAT) was performed.

### Plasma samples

Preoperative plasma samples were collected prospectively from an independent cohort of 20 patients suffering from cholangiocarcinoma who underwent surgery between November 2013 and September 2016 at the Department of Surgery, University of Bonn. Tumor staging and documentation of relevant data was performed as mentioned above. Plasma samples from 100 gender- and age-matched patients who did not have a history of a malignant tumor served as controls. Written consent was obtained from all patients.

### Preparation of sample DNA

For DNA extraction, bisulfite conversion and subsequent purification of genomic DNA from FFPE tissue biopsies and plasma samples, the innuCONVERT Bisulfite All-In-One Kit as well as the innuCONVERT Bisulfite Body Fluids Kit (Analytik Jena AG, Jena, Germany) were used, respectively. All kits were applied according to the manufacturer’s protocol.

### Real-time PCR quantification of *SHOX2* and *SEPT9* DNA methylation

Relative DNA methylation of the *SHOX2* and *SEPT9* locus was quantified by a methylation-specific triplex qPCR assay as previously described [39]. Methylation status of the *ACTB* locus served as the reference. Oligonucleotides used in the assay are summarized in Additional file 1: Table S2.

The *SHOX2* Amplicon is a 112 bp sequence and contains 11 CpGs. Four CpGs are included in the detection probe and three in the reverse primer. The *SEPT9* Amplicon is 60 bp long and contains five CpGs, which are all covered by the blocking primer. Additionally, three out of five CpGs are covered by the detection probe. Twenty-five nanograms of template DNA was used as template DNA for each single PCR reaction. PCR was carried out using an Applied Biosystems 7500 Fast Real-Time PCR System (Life Technologies Corporation, Carlsbad, CA, USA) and the following temperature profile was applied: 20 min at 95 °C followed by 50 cycles with 2 s at 62 °C, 45 s at 56 °C (each at 100% ramp rate), and 15 s at 95 °C (at 75% ramp rate) [39].

### Data analyses

The calibrator and all samples were measured in triplicate, and a mean average of the CT values was calculated. The ∆∆CT method was used to determine a relative methylation value for each valid sample as previously described: ∆∆CT_Sample_ = ∆CT_Sample_ − ∆CT_Calibrator_, where ∆CT_Sample_ = CT_Sample/Methylationquantificationassay_ − CT_Sample/Totalquantificationassay_ and ∆CT_Calibrator_ = CT_Calibrator/Methylationquantificationassay_ − CT_Calibrator/Totalquantificationassay_ [55, 56]. Percent methylated reference (PMR) values were calculated applying the following formula: Methylation_Sample_ = 100% × 2^−∆∆CT^ [56]. Quantitative *SHOX2* and *SEPT9* DNA methylation values were dichotomized by setting a methylation cut-off. The cut-off was set in order to reduce the false-positive rate for benign controls to 0% in tissue specimens and to 1% in plasma specimens. All samples with PMR values above the cut-off were classified as positive, while samples with methylation values below the cut-off were classified as negative.

### Statistical analyses

Statistical analysis was performed using the SPSS software version 22 (IBM, Armonk, NY, USA) and GraphPad Prism 6 (GraphPad Software Inc., San Diego, CA, USA).

Mann-Whitney *U* test was applied to test for differences in DNA methylation levels between the two groups. Bonferroni correction was used in case of multiple pairwise comparisons. Pearson’s correlation between *SHOX2* and *SEPT9* methylation was analyzed. Receiver operating characteristics (ROC) curves were constructed to investigate the ability of DNA hypermethylation to differentiate between samples from BTC patients and samples from patients without BTC. The sum of *SHOX2* and *SEPT9* DNA methylation values was used to calculate ROC curves for performance evaluation of the combined biomarker panel. The area under the ROC curve (AUC) represents the ability of the test to distinguish between patients with and without BTC.

Overall survival was defined as the time between surgery and death or date of last patient contact and used as primary endpoint for outcome analysis. Univariate and multivariate Cox proportional hazard model was applied to test the association between DNA methylation, clinicopathological parameters, and overall survival. *p* values refer to Wald test. Two-sided *p* values <0.05 were considered as statistically significant.

## Results

### Results from tissue analyses

#### Patients and tumors

Tissue samples from 71 biliary tract cancers including 42 male (59%) and 29 female (41%) patients with a median age of 63 years (range 36–83) were analyzed (clinical and pathological characteristics are listed in Table 1). Cholangiocarcinomas outnumbered gallbladder carcinomas (*n* = 54, 76% vs. *n* = 17, 24%). The majority of cholangiocarcinomas were IHC (*n* = 25, 35%). Only five tumors (7%) were well differentiated (G1), while the remaining were graded as moderately (G2) or poorly differentiated (G3) (*n* = 33, 46.5% each). Most BTC were locally advanced and classified as T3 (*n* = 38, 53.5%). Positive regional lymph nodes were found in 16 patients (22.5%), while intrahepatic metastases were present in six patients upon final histology (M1; 8.5%). Accordingly, the majority of tumors was staged as UICC III (*n* = 28, 39%). R0-resection was achieved in most of patients (*n* = 52, 73%).

**Table 1:** Clinicopathological data of 71 biliary tract cancer patients included in the tissue study. Associations of clinicopathological data with relative DNA methylation of *SHOX2* and *SEPT9* in tumor tissue

			*SHOX2* methylation	*SEPT9* methylation
Clinicopathological data	*n*	(%)		
			*p* value (*n*)	*p* value (*n*)
Tumor location				
GBC	17	(24)		
IHC	25	(35)		
PHC (Klatskin tumor)	22	(31)		
DC	7	(10)		
GBC vs. CC			0.46 (55)	0.014* (55)
Gender				
Male	42	(59)		
Female	29	(41)		
Male vs. female			0.10 (55)	0.14 (55)
Age at diagnosis				
≤60 years	31	(44)		
>60 years	40	(56)		
Median age [years]	63	NA		
Mean age [years]	62	NA		
Range [years]	36–83	NA		
≤60 vs. >60 years			0.57 (55)	0.031* (55)
Tumor grade				
G1	5	(7)		
G2	33	(46.5)		
G3	33	(46.5)		
G1, G2, vs. G3			0.22 (55)	0.68 (55)
Tumor stage				
T1	10	(14)		
T2	17	(24)		
T3	38	(53.5)		
T4	6	(8.5)		
T1, T2 vs. T3, T4			0.51 (55)	0.013* (55)
Lymph node status				
N0	32	(45.1)		
N1	15	(21.1)		
N2	1	(1.4)		
Unknown	23	(32.4)		
N0 vs. N1, N2			0.19 (39)	0.30 (39)
Venous invasion				
V0	42	(59)		
V1	19	(27)		
Unknown	10	(14)		
V0 vs. V1			0.97 (48)	0.76 (48)
Lymphatic invasion				
L0	34	(48)		
L1	20	(28)		
Unknown	17	(24)		
L0 vs. L1			0.67 (42)	0.70 (42)
Perineural invasion				
Pn0	17	(24)		
Pn1	35	(49)		
Unknown	19	(27)		
Pn0 vs. Pn1			0.37 (41)	0.82 (41)
Distant metastases				
M0	65	(91.5)		
M1	6	(8.5)		
M0 vs. M1			0.32 (55)	0.36 (55)
UICC stage				
UICC I	4	(6)		
UICC II	9	(13)		
UICC III	28	(39)		
UICC IV	10	(14)		
Unknown	20	(28)		
UICC I, II vs. UICC III, IV			0.08 (41)	0.06 (41)
Surgical margin				
R0	52	(73)		
R1	19	(27)		
R0 vs. R1			0.24 (55)	0.66 (55)
Follow-up				
Follow-up available	71	(100)		
Median follow-up [months]	15	NA		
Mean follow-up [months]	23	NA		
Range [months]	0–104	NA		
Deceased	50	(70)		
Censored	21	(30)		

*p* values refer to the Mann-Whitney *U* test

*p < 0.05

#### DNA methylation levels of *SHOX2* and *SEPT9*

**Fig. 1:**
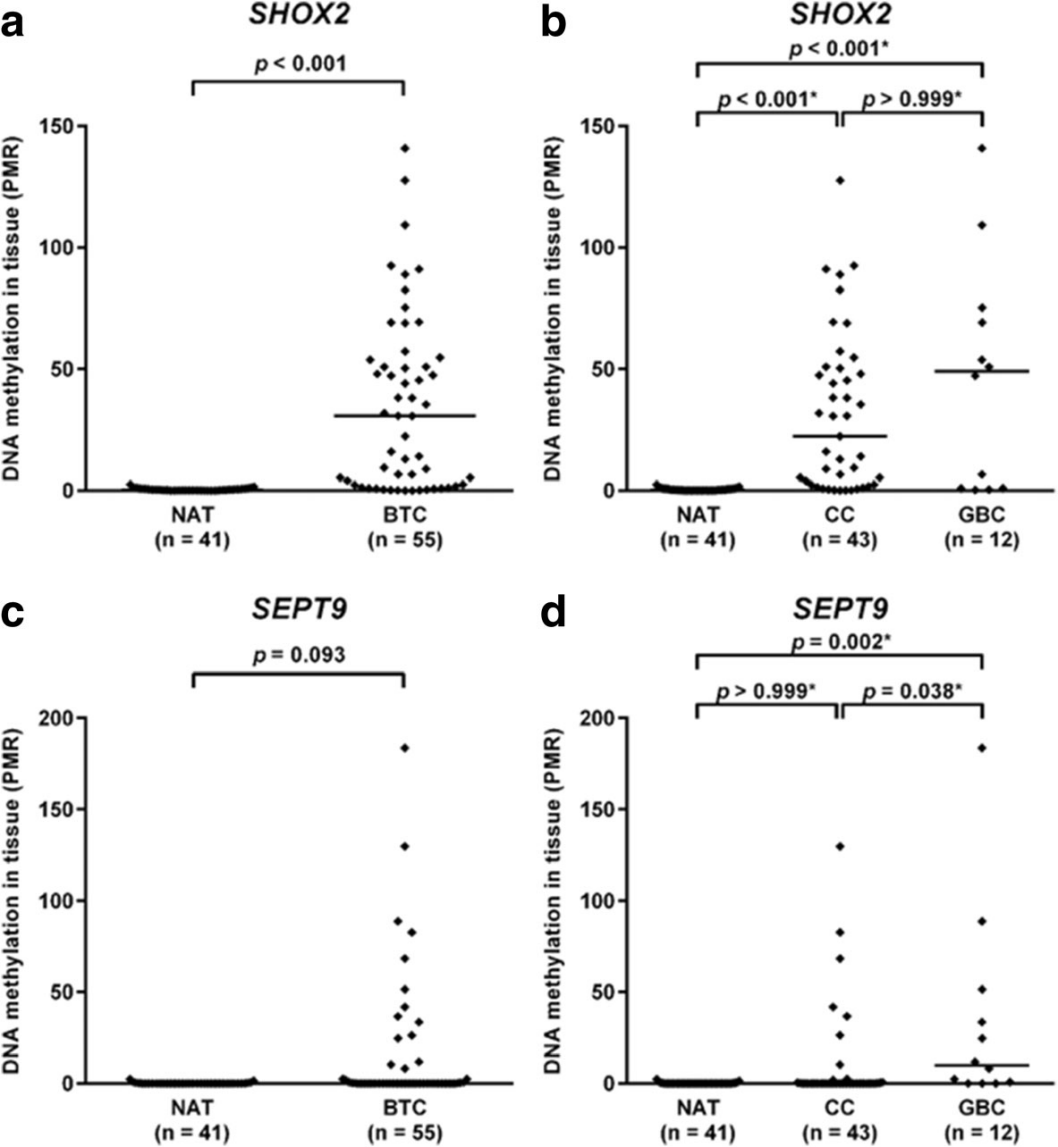
*SHOX2* and *SEPT9* DNA methylation levels in tissue samples. Relative DNA methylation values (PMR in %) of *SHOX2* are depicted as *black rhombuses* for **a** NAT and BTC specimens, as well as for **b** NAT, CC, and GBC specimens. PMR values of *SEPT9* are also depicted for **c** NAT and BTC specimens, as well as for **d** NAT, CC, and GBC specimens. *Horizontal lines* indicate median PMR values of sample series. *p* values refer to Mann-Whitney *U* test. *Bonferroni corrected *p* values for multiple pairwise compare

Percent methylated reference (PMR) values of the *SHOX2* and *SEPT9* gene obtained by quantitative methylation-specific real-time PCR are illustrated in Fig. 1. Valid measurements were obtained from 55 BTC and 41 NAT specimens. The performance of both methylation markers in differentiating tumor tissue from NAT was evaluated in all 55 BTC specimens collectively, as well as in CC (*n* = 43) and GBC (*n* = 12) individually. Background methylation with a maximum of 2.52% (*SHOX2*) and 2.32% (*SEPT9*) was present in most NAT samples, thus necessitating cut-offs for both gene loci to classify samples as either methylated or unmethylated.

*SHOX2* showed significantly higher DNA methylation values in BTC specimens and both subpopulations compared to NAT (Fig. 1a, b). Among 55 BTC specimens, 39 were correctly rated as *SHOX2* positive, resulting in a sensitivity of 71% with 100% specificity. *SHOX2* methylation frequencies in CC and GBC were 72 and 67%, respectively. No significant difference in *SHOX2* methylation levels was observed between CC and GBC (Fig. 1b).

Elevated *SEPT9* DNA methylation levels were also found in BTC specimens compared to NAT. However, the results were not statistically significant (Fig. 1c). Sixteen BTC samples showed *SEPT9* positivity, providing a sensitivity and specificity of 29 and 100%, respectively. If analyzed separately, however, carcinomas of the gallbladder presented significantly higher *SEPT9* methylation levels than both NAT and CC (Fig. 1d). While the methylation frequency of *SEPT9* reached only 19% in CC, it was as high as 67% in GBC.

**Fig. 2:**
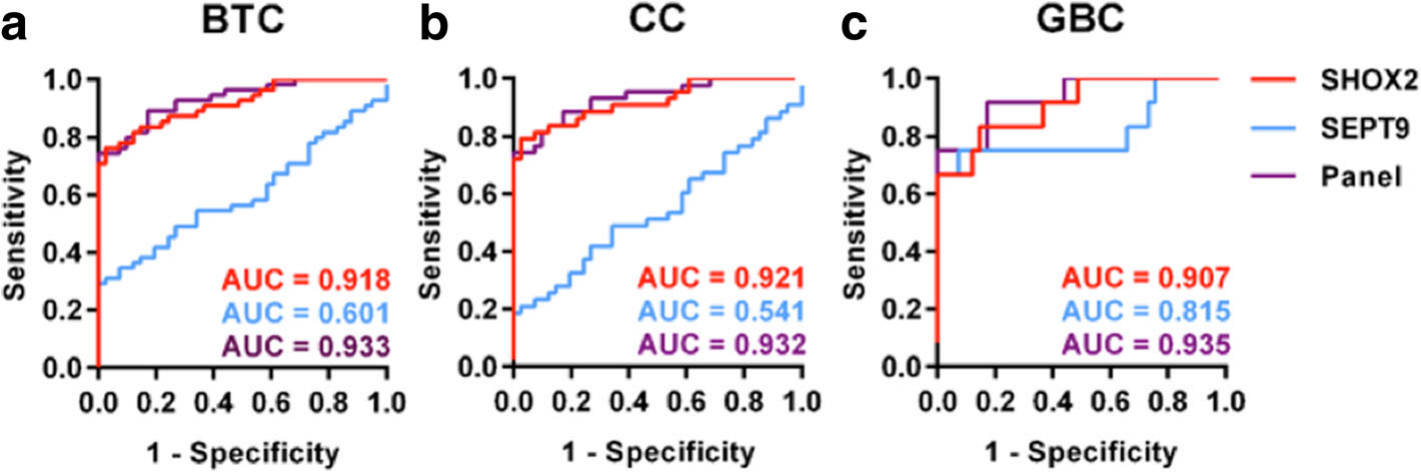
Receiver operating characteristic (ROC) curves for *SHOX2*, *SEPT9*, and the combined biomarker panel (based on the sum of PMR values) in tissue specimens. The ROC curves and resulting area under the curve (AUC) values are depicted for **a** all 55 BTC specimens: AUC—*SHOX2* = 0.918 [95% CI 0.865–0.971]; AUC—*SEPT9* = 0.601 [95% CI 0.488–0.713]; AUC—Panel 0.933 [95% CI 0.886–0.979] **b** 43 CC specimens: AUC—*SHOX2* = 09.21 [0.863–0.979]; AUC—*SEPT9* = 0,.541 [95% CI 0.416–0.666]; AUC—Panel = 0.932 [95% CI 0.880–0.984] and **c** 12 GBC specimens: AUC—*SHOX2* = 0.907 [95% CI 0.808–1.005]; AUC—*SEPT9* = 0.815 [95% CI 0.637–0.993]; AUC—Panel = 0.935 [95% CI 0.855–1.015], respectively

In a further step, the combined performance of *SHOX2* and *SEPT9* as a biomarker panel was evaluated. This panel was considered positive if at least one of both genes displayed DNA hypermethylation. Since high methylation values of *SHOX2* did not correlate significantly with *SEPT9* hypermethylation (Pearson’s correlation coefficient *r* = 0.24, *p* value = 0.08), the combined panel was expected to detect more cancer specimens than *SHOX2* or *SEPT9* alone. As anticipated, the panel correctly identified 75, 74, and 75% of BTC, CC, and GBC specimens, respectively. All 41 NAT samples were rated as *SHOX2* and *SEPT9* negative, resulting in a specificity of 100%. The ROC curves and AUC values for *SHOX2*, *SEPT9*, and the combined panel in BTC as well as in CC and GBC specimens are illustrated in Fig. 2.

Three BTC samples showed relative *SHOX2* DNA methylation values above the theoretically expected maximum of 100% (109.2, 127.6, and 140.8%). Similarly high *SEPT9* methylation levels were also found in two other cases (129.7 and 183.8%). Relative *SHOX2* and *SEPT9* DNA methylation values were determined by a triplex assay, which included a reference PCR of the ACTB gene to quantify the total amount of DNA. PMR values >100% have been previously reported for *SHOX2* in lung cancer, when *ACTB* was used as a reference, and were attributed to amplification of the *SHOX2* locus or deletion of the *ACTB* locus [34, 36, 56]. Therefore, we presume that deletion of the *ACTB* locus or amplification of the *SHOX2* and *SEPT9* locus, respectively, may have also led to PMR values >100% in these five BTC samples.

#### Association of DNA methylation with clinicopathological parameters

Elevated *SEPT9* methylation levels correlated significantly with advanced size of the primary tumor (T1/T2 vs. T3/T4, *p* = 0.013) as well as patient age (≤60 years vs. > 60 years, *p* = 0.031) (Table 1). However, no significant association was found between *SHOX2* or *SEPT9* methylation and the remaining clinicopathological parameters.

#### Survival analyses

Data on overall survival were available for all 71 patients. Median follow-up was 15 months (range 0–104 months), and 50 patients (70%) deceased during the follow-up time. Results from uni- and multivariate Cox proportional hazards analyses are shown in Table 2. Univariate survival analysis revealed that lymph node involvement (*p* = 0.031, HR = 2.2), lymphatic invasion (*p* = 0.007, HR = 2.4), and surgical margin status (*p* < 0.001, HR = 3.5) were of prognostic significance. On multivariate analysis merely microscopic residual carcinoma at the final surgical margin (R1) proved to be an independent prognostic factor associated with a dismal outcome (*p* = 0.026, HR = 3.8). DNA methylation status of *SHOX2* and *SEPT9* in tumor tissue showed no correlation with overall survival.

**Table 2:** Results from univariate and multivariate survival analyses (Cox proportional hazard models)

		Number of patients	Hazard ratio [95% CI]	*p* value (Wald test)
Univariate analysis				
Tumor location	(GBC vs. CC)	71	0.9 [0.5–1.8]	0.83
Gender	(Male vs. female)	71	1.4 [0.8–2.5]	0.22
Age at diagnosis	(≤60 vs. >60 years)	71	1.1 [0.6–2.0]	0.72
Tumor grade	(G1, G2 vs. G3)	71	1.6 [0.9–2.7]	0.13
Tumor stage	(T1, T2 vs. T3, T4)	71	1.0 [0.6–1.8]	0.94
Lymph node status	(N1, N2 vs. N0)	48	2.2 [1.1–4.5]	0.031*
Venous invasion	(V0 vs. V1)	61	1.3 [0.7–2.5]	0.42
Lymphatic invasion	(L1 vs. L0)	54	2.4 [1.3–4.6]	0.007*
Perineural invasion	(Pn0 vs. Pn1)	52	1.3 [0.6–2.5]	0.50
Distant metastases	(M0 vs. M1)	71	2.4 [0.9–6.1]	0.08
UICC stage	(UICC I, II vs. UICC III, IV)	51	1.0 [0.5–2.0]	0.91
Surgical margin	(R1 vs. R0)	71	3.5 [1.8–6.7]	<0.001*
*SHOX2* methylation^a^		55	1.0 [1.0–1.0]	0.52
*SEPT9* methylation^a^		55	1.0 [1.0–1.0]	0.78
*SHOX2* methylation	(*SHOX2*− vs. * SHOX2*+)	55	1.3 [0.6–2.6]	0.51
*SEPT9* methylation	(*SEPT9*− vs. *SEPT9*+)	55	1.1 [0.6–2.4]	0.72
Multivariate analysis				
Lymph node status	(N0 vs. N1, N2)		1.5 [0.6–3.5]	0.36
Lymphatic invasion	(L0 vs. L1)	34	2.1 [0.9–5.3]	0.11
Surgical margin	(R0 vs. R1)		3.8 [1.2–12.2]	0.026*

*SHOX2* and *SEPT9* DNA methylation levels were analyzed as continuous and dichotomized variables. *p* values refer to the Wald test

*p < 0.05

^a^Continuous variable

### Results from plasma analyses

**Fig. 3:**
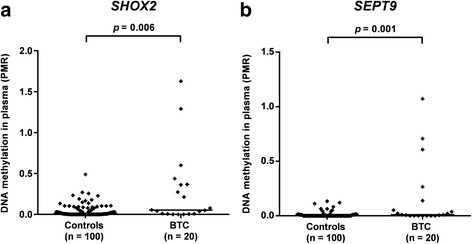
Relative DNA methylation values (PMR in %) of **a** *SHOX2* and **b** *SEPT9* in plasma samples from 100 controls and 20 cholangiocarcinoma patients. Each *black rhombus* represents a patient sample. *Horizontal lines* indicate median PMR values of sample series. *p* values refer to Mann-Whitney *U* test

DNA from plasma samples taken preoperatively from 20 cholangiocarcinoma patients, and 100 matched controls was analyzed for relative *SHOX2* and *SEPT9* DNA methylation (see Additional file 2: Table S1 for clinicopathological characteristics of both cohorts). Valid measurements were obtained from all 120 plasma samples, and respective PMR values are shown in Fig. 3. As in tissue analyses, detectable background methylation in samples from benign controls required a cut-off for dichotomization.

*SHOX2* DNA methylation was significantly higher in plasma specimens from CC patients than from controls and correctly identified 35% (7/20) of carcinoma cases (Fig. 3a). One control sample was falsely classified as *SHOX2* positive, resulting in 99% specificity. The area under the curve (AUC) was 0.691 (95% CI 0.554–0.828).

Similarly, plasma samples from patients with CC showed increased PMR values of the *SEPT9* gene locus (Fig. 3b). Five plasma samples showed *SEPT9* methylation values above the cut-off, resulting in a sensitivity of 25% with 99% specificity and an AUC of 0.699 (95% CI 0.561–0.837).

**Fig. 4:**
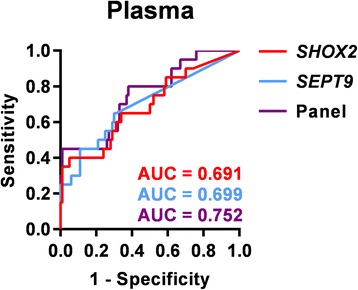
Receiver operating characteristic (ROC) curves and area under the curve (AUC) values for *SHOX2*, *SEPT9*, and the combined biomarker panel, based on the sum of PMR values, in plasma samples. AUC—*SHOX2* = 0.691 [95% CI 0.554–0.828]; AUC-*SEPT9* = 0.699 [95% CI 0.561–0.837]; AUC-Panel = 0.752 [95% CI 0.631–0.873]

As in tumor tissue, no correlation between *SHOX2* and *SEPT9* DNA methylation was observed (Pearson’s correlation coefficient *r* = 0.072, *p =* 0.76). Therefore, *SHOX2* and *SEPT9* as a panel displayed a higher sensitivity than both gene loci. Nine out of 20 cholangiocarcinoma cases (45%) were detected, while 99% of control samples were accurately classified as *SHOX2* and *SEPT9* negative. The combined biomarker panel achieved an area under the curve (AUC) of 0.752 (95% CI 0.631–0.873). The ROC curves and AUC values of *SHOX2*, *SEPT9*, and the combined panel in plasma samples from cholangiocarcinoma patients are shown in Fig. 4

## Discussion

Epigenetic alterations such as aberrant DNA methylation patterns have been extensively studied across multiple human cancer types, with BTC being no exception. Genome-wide methylation analyses in CC revealed frequent promoter hypermethylation of homeobox genes, a gene class known to be involved in various developmental processes [17, 18]. Recently, Goeppert et al. published global methylation data for intrahepatic and extrahepatic CC and identified numerous aberrantly methylated genes involved in cholangiocarcinogenesis, including components of the Wnt signaling pathway [19]. Unlike the above referenced, the majority of studies has focused on the methylation status of a few specific gene loci and their capability to distinguish between malignant and benign tissues, biliary brush cytologies, and bile juice specimens [16, 20–28]. In the present study, a similar approach was followed by selecting two candidate genes for methylation analysis in BTC, namely *SHOX2* and *SEPT9*, which have been reported to be aberrantly methylated in other tumor entities. Due to afore evidenced clinical implications, we sought to evaluate the methylation status of *SHOX2* and *SEPT9* and their potential as biomarkers in tissue and plasma specimens from BTC patients.

To our knowledge, this is the first report of recurrent *SHOX2* hypermethylation in BTC. *SHOX2* methylation status has been previously investigated in other malignant tissue specimens with varying results. Ninety-six percent of lung cancer samples showed more pronounced methylation than matched NAT samples from the same patients [34]. Merely 36% of papillary thyroid carcinomas were found to be methylated at the *SHOX2* locus [57]. We observed a *SHOX2* hypermethylation in 71% of BTC specimens while no normal tissue sample harbored this alteration emphasizing the malignant association.

*SEPT9*, as part of a four genes panel, has been recently evaluated as a biomarker for CC in tumor tissue as well as in biliary brush samples [16]. In tissue specimens, *SEPT9* was described to differentiate between CC and non-malignant controls with a sensitivity of 26% and a specificity of 100% (AUC = 0.63) [16]. Our study supported these results, although we observed *SEPT9* to be slightly less sensitive in CC. This is possibly due to selection bias in relatively small cohorts of rare entities. While our samples solely comprised formalin-fixed and paraffin-embedded tissue, Andresen et al. included fresh frozen tissue samples, too [16]. In a previous study using the same CC series, both sample materials were compared and methylation frequencies of *CDO1*, *ZSCAN18*, *SFRP1*, and *DCLK1* were found to be generally lower in FFPE specimens than in the fresh frozen sample set while modern sensitive techniques may overcome this observation [29].

In our study, we demonstrated that the DNA methylation of *SHOX2* and *SEPT9* can accurately differentiate between tumor tissue from BTC patients and normal adjacent tissue with high specificity. However, the performance of both markers varied markedly in terms of sensitivity, and *SHOX2* outperformed *SEPT9* in detecting BTC by more than double. The results confirm our hypothesis that epigenetic alterations of *SHOX2* and *SEPT9* may serve as biomarkers to support the detection of BTC in tissue specimens due to its high specificity, and a use as biomarkers for response prediction can be discussed. However, the application of this methylation panel test in the clinical setting as a diagnostic tool may be limited because of its limited sensitivity. In order to overcome the low sensitivity, other biomarkers could be added to the panel. A recent study identified DNA methylation level of *OPCML* and *SFRP1* as a potential diagnostic biomarker in BTC [58]. The AUC value of *OPCML* was 0.932 and of *SFRP1* was 0.951. The sensitivity and specificity of *OPCML* were 89 and 100%, respectively, and of *SFRP1* were 83.6 and 85.5%. The analysis of a combined methylation panel including *SFPR1*, *OPCML*, *SHOX2*, and *SEPT9* in plasma could increase the diagnostic value of the methylation panel.

The fact that *SHOX2* and *SEPT9* show different promoter methylation in BTC plasma specimens also suggests a clinical application for minimally invasive diagnosis in various clinical samples. Half a century after Mendel and Metais discovered the presence of cell-free nucleic acids in human blood, it has become generally acknowledged that extracellular DNA from cancer patients also harbors tumor-related epigenetic alterations [59]. Accordingly, aberrant methylation of circulating cell-free DNA has been extensively studied as a biomarker in human cancer [60]. To our knowledge, larger studies on quantification of methylated circulating DNA in BTC are thus far not published. Herein, we report for the first time that altered promoter methylation can also be detected in plasma from patients with cholangiocarcinoma. *SHOX2* and *SEPT9* DNA methylation levels showed a high specificity in differentiating between plasma from CC patients and controls. However, methylation analysis of *SHOX2* and *SEPT9* in plasma, naturally containing circulating DNA from any site of the body, bears some disadvantages for diagnostic purposes in BTC. In fact, the amount of methylated DNA derived from the biliary tract is presumably low in proportion to total sample DNA. This could explain the limited sensitivity observed in our study. The addition of other established biomarker like CA19-9 to this panel could increase the test sensitivity. This issue should be investigated in future prospective studies in order to asses the value of this methylation panel as a part of a more complex diagnostic tool.

Interestingly, aberrant DNA methylation patterns have been observed in bile fluid as well as in biliary brush cytology specimens and were described to be capable of differentiating between BTC and benign controls [16, 25–28]. *SEPT9* has recently been reported to achieve a sensitivity of 57% with 100% specificity in biliary brush samples from CC patients, while respective tissue samples were methylated in less than 30% [16]. In our tissue study, *SHOX2* methylation proved to be superior to *SEPT9* in terms of sensitivity to correctly identify BTC specimens. Therefore, it seems reasonable to suggest that *SHOX2* DNA methylation could also serve as biomarker for BTC in biliary brush samples or bile fluids. Andresen et al. investigated a four-gene methylation biomarker panel (*CDO1*, *CNRIP1*, *SEPT9*, and *VIM*) in biliary brush sample of patients with CC. This test achieved a 85% sensitivity and a 98% specificity. Thus, future investigations should analyze the role of a five-gene panel including *CDO1*, *CNRIP1*, *VIM*, *SEPT9*, and *SHOX2* as a diagnostic tool in plasma from patients with BTC.

In our cohort, residual carcinoma at the final histological margin proved to be the only independent predictor of survival. This finding is in line with previous studies linking R1-resection to an unfavorable outcome in BTC [61–63]. Methylation status of *SHOX2* and *SEPT9* showed no relation to overall survival in our cohort. Nevertheless, a prognostic value of methylated *SHOX2* and *SEPT9* has been reported in the literature, although the findings were partially ambiguous. For instance, low *SHOX2* DNA methylation values predicted a shorter progression-free survival following resection in non-small cell lung cancer, whereas hypermethylation of *SHOX2* in pleural effusions was associated with an adverse outcome [39, 56]. Schmidt et al. recently proposed quantitative measurement of methylated *SHOX2* DNA in plasma as a useful tool to monitor therapy response in advanced stage lung cancer patients [36]. Furthermore, an increased methylation level of *SEPT9* was a predictor of poor prognosis in esophageal squamous cell carcinoma, as well as in prostate cancer on androgen deprivation [52, 53]. Likewise, colorectal cancer patients with high *SEPT9* serum methylation levels at 1-year follow-up were found to be at high risk of disease recurrence [64]. Although these promising findings in other cancer entities suggested that aberrant *SHOX2* and *SEPT9* methylation may also correlate with prognosis in BTC, our results did not confirm this hypothesis. This could be attributed to our relatively limited sample number. Therefore, we recommend further research on the prognostic value of *SHOX2* and *SEPT9* DNA methylation in a larger cohort of BTC patients.

Our study has some limitations: Due to the rareness of BTC, we only included a limited number of patients, and CCs were not subgrouped into IHC, PHC, and DC though distinct differences in methylation profiles have been observed between intrahepatic and extrahepatic CC [22, 23]. Moreover, a subgroup analysis of clinicopathological data and methylation status separately for CC and GBC was not performed. The subgroups were not large enough in order to obtain a sufficient statistical power. Therefore, we did not perform an association analysis for CC and GBC. Thus, a larger study cohort, possibly based on a multicenter approach in a prospective fashion, is needed to more precisely assess the potential of *SHOX2* and *SEPT9* methylation as a biomarker for all four subgroups of biliary tract cancer. Another limitation of our study is the lack of a targeted sequencing of *SHOX2* and *SEPT9*. This could add some important information concerning the promoter methylation status of both genes. Thus, we cannot provide information about the methylated CpG sites in tumor and normal tissue. Wasserkort et al. already demonstrated that hypermethylation of *SEPT9* in adenoma and CRC specimens is limited to one of the several CpG islands [47]. Moreover, investigation of intragenic CpGs and alternative promoters could add valuable information about these two genes. Therefore, a targeted sequencing should be performed in further studies in order to clarify the methylation status of *SHOX2* and *SEPT9* in cancer.

## Conclusions

Biliary tract cancer remains a fatal disease; thus, novel molecular markers enabling early diagnosis, treatment implementation, and therapy monitoring are urgently needed. Epigenetic alterations are promising for the development of biomarkers in human cancer. In this study, we showed that the *SHOX2* gene locus is frequently methylated in BTC. In addition, our results confirmed the presence of aberrant methylation of the *SEPT9* locus in BTC. Together, both markers identified 75% of BTC specimens with 100% specificity. Furthermore, we demonstrated that methylated *SHOX2* and *SEPT9* DNA can be detected in plasma from BTC patients highlighting a use in clinical samples for minimally invasive diagnostic applications and therapy monitoring. Future studies are needed to validate the biomarker capabilities of *SHOX2* and *SEPT9* in BTC before a possible establishment in clinical practice.

## Additional files

Additional file 1: Table S2. Primers locations and sequences. (DOCX 181 kb)
Additional file 2: Table S1. Clinicopathological data of the 20 biliary tract cancer cases and 100 gender- and age-matched controls included in plasma study. (XLSX 116 kb)

## Abbreviations

AJCC: American Joint Committee on Cancer
AUC: Area under the ROC curve
BTC: Biliary tract carcinoma
CC: Cholangiocarcinoma
CEA: Carcinoembryonic antigen
CRC: Colorectal cancer
DC: Distal cholangiocarcinoma
EBUS-TBNA: Endobronchial ultrasound with transbronchial needle aspiration
ERC: Endoscopic retrograde cholangiography
EUS-FNA: Endoscopic ultrasonography fine needle aspiration
FFPE: Formalin-fixed paraffin-embedded
GBC: Gallbladder carcinoma
IHC: Intrahepatic cholangiocarcinoma
NAT: Normal adjacent tissue
PCR: Polymerase chain reaction
PHC: Perihilar cholangiocarcinoma
PMR: Percent methylated reference
ROC: Receiver operating characteristic
