# Supplementary material for: Promoter hypermethylation of SHOX2 and SEPT9 is a potential biomarker for minimally invasive diagnosis in adenocarcinomas of the biliary tract
Source: Clin Epigenetics. 2016 Dec 12;8:133. doi: 10.1186/s13148-016-0299-x (PMC5153824; doi:10.1186/s13148-016-0299-x)
Supplement: Additional file 1: Table S2. — Primers locations and sequences. (DOCX 181 kb) [file 13148_2016_299_MOESM1_ESM.docx]

Additional file 1: Table S2: Primer Location and Sequences.

| **Primer** | **Sequence 5’-->3’** |
| --- | --- |
| *SHOX2* |  |
| chr3:157821339-157821449 |  |
| Forward Primer | GTTTTTTGGATAGTTAGGTAAT |
| Forward Blocker | TAATTTTTGTTTTGTTTGTTTGATTGGGGTTGTATGA-Spacer C3 |
| Reverse Primer (MSP-Primer) | TAACCCGACTTAAACGACGA |
| Hydrolysis Probe | 6-FAM-CTCGTACGACCCCGATCG-BBQ-650 |
|  |  |
| *SEPT9* |  |
| chr17:75369563-75369622 |  |
| Forward Primer | AAATAATCCCATCCAACTA |
| Reverse Primer | GTTGTTTATTAGTTATTATGT |
| Reverse Blocker | GTTATTATGTTGGATTTTGTGGTTAATGTGTAG-Spacer C3 |
| Hydrolysis Probe | JOE-TTAACCGCGAAATCCGAC-BHQ1 |
|  |  |
| *ACTB* |  |
| chr7:5571731-5571859 |  |
| Forward Primer | GTGATGGAGGAGGTTTAGTAAGTT |
| Reverse Primer | CCAATAAAACCTACTCCTCCCTTAA |
| Hydrolysis Probe | Cy5-ACCACCACCCAACACACAATAACAAACACA-BBQ650 |
